# Emerging neglected helminthiasis and determinants of multiple helminth infections in flood-prone township in Myanmar

**DOI:** 10.1186/s41182-018-0133-6

**Published:** 2019-01-04

**Authors:** Kay Thwe Han, Khin Thet Wai, Kyin Hla Aye, Khine Wah Kyaw, Wai Phyo Maung, Tin Oo

**Affiliations:** Parasitology Research Division, Department of Medical Research (DMR), No. 5 Ziwaka Road, Yangon, 11191 Myanmar

**Keywords:** Neglected helminthiasis, Determinants, Community risk groups, Myanmar

## Abstract

**Background:**

Myanmar has similar agro-based ecology and environmental risks as others in the Greater Mekong sub-region leading to the broad array of helminthic infections. Basic health staff (BHS) from the public sector forms a key stakeholder group in deworming interventions. The study aimed to ascertain the prevalence and determinants of multiple species helminth infections to promote township-level integrated interventions.

**Methods:**

A cross-sectional implementation research study in 2017 covered randomly selected 240 households in four villages of Shwegyin Township. Trained interviewers administered the pre-tested structured questionnaire to either the household head or the assigned person concerning their knowledge, perceptions, practices, food habits, and deworming experience. Concomitantly, the research team collected a single stool sample from each of 698 participants (age range of 8 months to 87 years) from 93% (224/240) of eligible households and examined by Kato-Katz smear microscopy. Eventually, 16 BHS joined the interactive dialogue session based on research evidence and knowledge translated for further validation.

**Results:**

The estimated prevalence of at least one helminth infection was 24% [168/698; 95% CI 21.0–27.0]. Apart from the soil-transmitted helminths (14%), zoonotic helminths especially *Taenia* spp. (0.7%) and *Schistosoma* spp. (3%) were detected. Almost half of the seasonally mobile gold panning workers (12/25; 48%) and 46% of pre-school-age children had helminth infections. Community risk groups at riverside villages had significantly higher multiple species helminth infection than those from inland villages (AOR = 10.9; 95% CI 4.9–24.2). Gold panning workers had higher infection rates than other categories (AOR = 2.5; 95% CI 0.6–9.5) but not significant. In flood-prone areas, householders failed to follow the guidelines to construct/re-construct specific type of sanitary latrines and challenges remained in disseminating health messages for community engagement. The innovative ideas recapitulated by BHS included the integration of health talks during the sessions for small agricultural loans and to harness advocacy with water, sanitation, and hygiene interventions.

**Conclusions:**

The emerging evidence of neglected zoonotic helminths required attention to introduce the periodic mopping-up and the “selective deworming plan” for vulnerable groups to cover the missed targets. Further multidisciplinary research to confirm the intermediate hosts and vectors of zoonotic helminths in the environment is essential for surveillance and response.

**Electronic supplementary material:**

The online version of this article (10.1186/s41182-018-0133-6) contains supplementary material, which is available to authorized users.

## Background

Neglected intestinal helminth infections affect marginalized, low-income, and resource-constrained regions among which more than 1.5 billion people or 24% of the world’s population are infected with at least one type of soil-transmitted helminth (STH) infections in 2016. However, the true burden of food-borne trematodiasis (FBT) and schistosomiasis in conjunction with STH is unclear [1, 2]. Recent updates in responses of health systems towards the disease burden of neglected helminth infections to improve disability-adjusted life years (DALYs) indicate preventive chemotherapy to reduce transmission. Nevertheless, immediate reinfections are not uncommon as well as multiple species infections and a myriad of factors are responsible such as poverty, poor sanitation, poor food hygiene, the presence of animal reservoirs in close proximity to communities, and cultural food habits [3]. A strategic plan of WHO (2012–2020) aims at the reduction of morbidity, elimination of heavy infections, and enhancement of control programs to achieve the transmission interruption of neglected helminth infections [4].

Myanmar has similar agro-based ecology and environmental risks as others in the Greater Mekong sub-region leading to the broad array of helminthic infections. Basic health staff (BHS) from the public sector forms a key stakeholder group in deworming interventions. Earlier studies in Myanmar reported the negative impact of STH on community health [5–7], and two studies had already documented the effectiveness of deworming on nutritional status [8–10]. The previous survey in Myanmar in 2012 estimated 30% of school children in the plains and 38% in the coastal area were infected with any of the STH parasites (*Ascaris lumbricoides*, *Trichuristrichiura*, and hookworm) [11, 12]. One collaborative study conducted in Shwegyin Township, Bago Region, from 2012 to 2014 reported that one-time routine examination of stool samples from 383 pregnant women at first antenatal visit could elicit 22.7% of STH infections, and ascariasis contributed 44.8%. Moreover, four cases of schistosomiasis, three cases of clonorchiasis, and two cases of fasciolopsiasis were detected among apparently healthy pregnant women [13]. Alongside favorable geographical and ecological circumstances, the distribution and burden of helminth infections reflect the level of political commitment and investment in resources for the prevention and control of helminthiases [14].

To date, public sector health services are mainly responsible for school-based deworming interventions and provision of albendazole together with diethylcarbamazine for mass drug administration campaigns in controlling filariasis in endemic regions of Myanmar [15]. Nevertheless, an inadequacy of current control efforts might lead to persistent hot spots at ecological niches and frequent reinfections remain as emerging and re-emerging public health challenges. Moreover, low level of correct knowledge of STH infections was reported by one study on school children and their parents/guardians in the central part of Myanmar [16]. The deeper understanding of the biological, environmental, and social determinants of helminth infections apart from other driving forces is critical by navigating an implementation research. Also, it is imperative to underscore the necessity for novel intervention tools that fit limited resources [17, 18]. Moreover, little is known about the magnitude and risk factors of STH, and zoonotic food-borne trematodiasis and schistosomiasis in Myanmar which needs further exploration to introduce integrated interventions in the vulnerable communities [19, 20]. Thus, baseline epidemiological information of these neglected parasitic diseases is crucial for timely action and surveillance among community risk groups. Therefore, this study aimed to ascertain three key deliverables: the magnitude of neglected helminth infections among all age groups, the contribution of risk factors for intestinal helminth infections in selected rural sites of Shwegyin Township, and the innovative strategies to leverage the formulation of a township-level integrated plan which are evidence-based and replicable to other vulnerable sites and endemic regions.

## Methodology

### Study design and duration

A cross-sectional implementation research study was conducted in Shwegyin Township, Bago-East Region, during 2017. The study was conducted from October 2016 to March 2017.

### Study area and study population

Shwegyin Township is located at latitude 17° 55′–14° 78′ (north) and longitude 96° 52′–32° 14′ (east) and is approximately 240 km north from Yangon City, a business capital of Myanmar (see Additional file 1). This township was purposely selected due to its annual flooding events that affected environmental sanitation and the existence of freshwater wetlands in proximity to the hydropower dam [19] apart from its popularity in fermented fish production and the predominance in consumption of raw fermented fish as the traditional dietary habit. Seasonal mobility is high in the township seeking for better economic opportunities. In addition to subsistence farming, small-scale gold mining is popular [21]. Shwegyin is included in the lower Sittaung river sub-basin area as indicated by Norwegian-Myanmar Bilateral Environment Programme in 2017, and the average annual rainfall is around 3000 mm. According to the 2014 Census, the total population of the township was 107,462 and 77% resided in rural areas [22]. The study population covered all householders in villages except under 6 months of age.

### Sample size determination and sampling procedure

The sample size of 235≈240 households met the assumptions of the 95% confidence level at 6% precision and the estimated prevalence of 40% of STH in households considering for 5% non-response. Following the two-way stratification procedure [23], two villages along the riverside and the equal number of villages from inland 4–6 km away from the Shwegyin River were purposively selected from the list prepared by the Township Health Department. Next, a total of 240 households (HH) (60 HH each from Ingani and Thanseik as inland villages and the equal number from Taungbat and Taguntaing as riverside villages) were planned to select at random. However, due to seasonal mobility, there were vacant households, especially in inland villages. Therefore, the lesser number of households was recruited in that villages compared to riverside villages (101 HH vs. 139 HH). All rural householders of both sexes with age above 6 months were invited to participate in the study.

### Data collection methods

The research team initiated the field survey after the annual flooding event around August 2017. A pilot survey was done to advocate the local health authorities and to pretest the structured interview questionnaire (SIQ). At the selected households (*n* = 240), the research team explained the study objectives, stool sample collection procedures, and risks and benefits of the study to all household members to attain the verbal informed consent for their voluntary participation. Then, trained interviewers administered the SIQ to the household head or the assigned person at and over 18 years of age focusing on their living conditions, age, gender, occupation, education, knowledge on intestinal helminths, food habits, deworming history, and hygienic and sanitation practices. The anonymity, privacy, and confidentiality issues were observed following the Helsinki Declaration during the structured interviews and stool collection.

### Stool sample collection

A dry wide-mouth sterile stool container was properly labeled (village, HH code, name, age, and sex) and delivered to all household members except children under 6 months of age. These stool containers were recollected in the next morning. The single stool sample was collected only taking into account feasibility under field conditions. After collecting the stool sample in the sterile container and during the transportation by cold chain to the township hospital, the required temperature was maintained. Of 240 households sampled, only 224 households (93%) provided stool samples though they had consented verbally. The stool samples were examined by two methods [direct wet mount examination and Kato-Katz thick smear method (K-K)] within 3 h of sample collection at the Township Hospital. Direct wet mount microscopic examination was done applying WHO reference method [24]. A clean slide was used to mix a stool equivalent to a match stick head (2 mg) with one drop of normal saline, and then, the wet mounts were examined under a light microscope with × 4, × 10, × 20, and × 40 magnifications.

For K-K stool smear examination, thick stool smear was prepared and a 3% methylene blue-glycerol solution-treated cellophane strip was applied according to the manufacturer’s instruction. Next, K-K thick smear examination was performed applying WHO guidelines [25].

To eliminate observer bias, two trained laboratory technicians examined the stool samples independently and the senior microbiologist made final validation of discordant smears between two technicians and also examined the randomly selected 5% of total smears [26].

### Data entry and analysis

The survey data were double entered in EPI Data entry software (version 3.1, Epidata Association, Odense-Denmark) for validity and analyzed by SPSS (version 22.0, IBM Corporation, New York, USA). Descriptive statistics were used to examine the social and demographic characteristics of the study population and the observed overall prevalence ratio and 95% confidence intervals (CI) of helminth infections. For multivariate analysis, an inclusion criterion of *P* < 0.2 was considered to select the variables. The major outcome variable was the presence of single or multiple species helminth infections. The multinomial logistic regression procedure (none detected vs. one species only; none detected vs. more than one species) was then employed to ascertain the adjusted odds ratios (AOR) and 95% CI of risk factors related to single and multiple species helminth infections while controlling for other variables. Further cross-tabulations were done, and the chi-square test was used to compare demographic characteristics, knowledge, experiences, perceptions, food habits, and hygiene and sanitation practices related to helminth infections between inland and riverside villages. *P* ≤ 0.05 was considered significant.

### The dissemination meeting

For sharing of salient findings and for validation of risk factors for helminthiasis by the key stakeholders responsible for deworming interventions, the dissemination meeting was held at the Township Health Department (THD) in December 2017. The evidence-based information from the completed survey as a trigger was conveyed to the 16 BHS of the health centers of the THD which was essential in reporting the implementation research studies. A two-way interactive dialogue session was used as a dissemination tool for implementation research [27] and facilitated by two members of the research team. The 2-h session confined third key deliverable in three components: age group and occupational-specific prevalence of helminth infections, variations in best practices in prevention by ecological setting, and the proposed strategic approaches to incorporate in the township-level integrated health plan.

## Findings

### Demographic, social, and household characteristics

The demographic and social characteristics were presented. The age group above 15 years contributed 65% of 698 study participants which was significantly higher in inland villages compared to riverside villages. And the age group less than 5 years (pre-school-age children) (Pre-SAC) was only 4%. The proportion of female was higher than that of male in the study households (55%), and over one third were students (37%). The age range of householders varied from 8 months to 87 years. Less than 60% of householders had the low level of education attainment. However, householders in riverside villages were significantly more likely to attain higher education level compared to inland villages (Table 1). The principal source of drinking water supply reported in 224 households was from the tube wells (44.8%) followed by public taps (23.8%), unprotected tube wells (15.9%), and protected brick wells (10.5%). The remaining 5% was rain water. Approximately, the household ownership of sanitary latrines was reported as 92% (206/224).

**Table 1:** Demographic and social characteristics of study participants in selected households in Shwegyin Township, Myanmar, 2017 (*n* = 698)

Characteristic	Inland villages		Riverside villages		Combined	
	Number	Percent	Number	Percent	Number	Percent
Age group*						
< 5 years (Pre-SAC)	0	0.0	11	2.8	11	1.6
5–14 years (SAC)	110	36.9	120	30.0	230	33.0
≥ 15 years	188	63.1	269	67.3	457	65.4
Gender						
Male	138	46.3	176	44.0	314	45.0
Female	160	53.7	224	56.0	384	55.0
Type of occupation						
Student	112	37.6	147	36.8	259	37.1
Others^Ϯ^	107	35.9	129	32.3	236	33.8
Dependent	46	15.4	53	13.3	99	14.2
Farmer	21	7.0	54	13.5	75	10.7
Gold panning worker	9	3.0	16	4.0	25	3.6
Fisherman	3	1.0	1	0.3	4	0.6
Highest education level attained*						
Not yet school going age	0	0.0	11	2.8	11	1.6
Illiterate and primary school	220	73.8	167	41.8	387	55.4
Middle school	60	20.1	132	33.0	192	27.5
High school and above	18	6.0	90	22.5	108	15.5

Zero cells were merged to compute chi-square test

*PRE-SAC* pre-school-age children, *SAC* school-age children

^Ϯ^Others include odd jobs and itinerant vendors

*P < 0.05

### Prevalence of helminth infections

The overall prevalence of study participants with at least one helminth infection by direct wet mount method was 14.5% [102/698; 95% CI 12.0–17.0] whereas the K-K method identified the higher prevalence of helminth infection as 24% [168/698; 95% CI 21.0–27.0]. And of whom 13.7% [41/298; 95% CI 8.0–15.0] was detected in inland villages and 33.9% [136/400; 95% CI 29.0–39.0] in riverside villages. Apart from STH (14%), zoonotic and food-borne trematodes especially *Taenia* spp. (0.7%) and *Schistosoma* spp. (3%) were detected. Almost half of the seasonally mobile gold panning workers (12/25; 48%) and 46% of pre-school-age children had helminth infections.

Table 2 showed the comparison of prevalence of helminth infections by two methods (direct wet mount and Kato-Katz). As might be expected, the K-K method could detect the higher prevalence estimates of all types of helminths per 1000 slides examined compared to the wet mount examination in both inland and riverside villages. The K-K method detected the higher rates of *Trichuris trichiura* and liver flukes compared to the wet mount method. By wet mount examination, there was a chance to miss the small-sized eggs like liver flukes and the egg counts were low and most of the participants were asymptomatic. The larvae of *Strongyloide*s *stercoralis* may be cut into pieces while sieving the stool samples in Kato-Katz smear preparation. Therefore, only wet mount examination could detect strongyloidiasis.

**Table 2:** Prevalence estimates of helminths infection per 1000 stool samples examined in Shwegyin township, Myanmar, stratified by location of villages and the type of laboratory method used, 2017

Helminth diversity	Prevalence estimates of helminths infection per 1000 stool samples examined			
	Inland villages (*n* = 298)		Riverside villages (*n* = 400)	
	Wet mount	Kato-Katz	Wet mount	Kato-Katz
*Ascaris lumbricoides*	67	70	189	199
*Trichuris trichiura*	1	33	142	211
Hookworm	30	30	40	42
*E. vermicularis*	3	10	10	10
*S. stercoralis*	20	0	12	0
*Taenia* spp.	7	10	2	5
*Schistosoma* spp.	7	20	15	42
*O. viverrani/C. sinensis* (liver fluke)	7	17	2	25
*Paragonimus* spp. (lung fluke)	0	0	15	20

### Factors associated with single and multiple species helminth infections

The ecological, biological, occupational, and environmental factors associated with multiple species helminth infections were analyzed (Table 3). Householders from villages along the riverside compared to those from inland villages were significantly more likely to be infected with both single species (AOR 3.1; 95% CI 1.7–5.5) or multiple species helminth infections (AOR 10.9; 95% CI 4.9–24.2). Thus, the location of villages had a significant impact on the prevalence of neglected intestinal helminth infections. Among the different categories of occupation, gold panning workers were more likely to be infected with multiple helminths compared to the remaining categories (AOR 2.5; 95% CI 0.6–9.5) but not significant probably due to the small sample (*n* = 25). However, helminth infection rates did not differ by specific age group and gender. The odds of multiple species helminth infections was 0.5 times significantly lesser in households with drinking water supply mainly from piped water whereas the type of flooring and the type of latrine available in the households did not significantly contribute for being infected with helminths. Moreover, the reported deworming within past 6 months did not have a significant effect on both single and multiple species helminth infections in the study population.

**Table 3:** Helminths infections and associated factors in Shwegyin Township, Myanmar, 2017 (*n* = 698)

Characteristic	Number	None detected (%)	One spp. (%)	> One spp. (%)	None vs. One spp.		None vs. > One spp.	
					AOR	95% CI	AOR	95% CI
Location								
Inland	298	84.2	12.1	3.7	1.0		1.0	
Riverside	400	63.3	20.5	16.3	3.1	1.7–5.5	10.9	4.9–24.2
Flooring								
Wood and cement	534	73.4	15.5	11.0				
Others	164	68.3	21.3	10.4				
Latrine								
Flush latrine	647	71.6	17.2	11.3				
None and insanitary latrine	51	80.4	13.7	5.9				
Gender								
Male	314	75.5	13.7	10.8				
Female	384	69.5	19.5	10.9				
Drinking water source								
Tube well	310	71.3	14.8	13.9	1.0		1.0	
Piped water	175	70.3	21.1	8.6	1.1	0.6–1.8	0.5	0.2–0.9
Dug well	213	75.1	16.4	8.5	1.9	1.1–3.5	2.0	0.9–4.3
Age group								
≥ 15 years	457	72.2	17.1	10.7	1.0		1.0	
5–14 years	230	73.0	15.7	11.3	0.8	0.4–1.8	0.9	0.3–2.5
< 5 years	11	54.5	36.4	9.1	2.2	0.6–8.0	0.9	0.1–8.5
Occupation								
Student	259	71.4	16.6	12.0	1.0		1.0	
Others	414	73.9	16.9	9.2	0.8	0.4–1.7	0.7	0.3–1.9
Gold panning	25	52.0	20.0	28.0	1.3	0.4–4.6	2.5	0.6–9.5
Deworming within past 6 months								
Yes	336	73.8	17.0	9.2	1.0		1.0	
No and DK	362	70.7	16.9	12.4	1.0	0.7–1.6	1.4	0.8–2.3

From the 701 study participants, two cases were truncated due to incomplete data

*AOR* adjusted odds ratio, *DK* do not know, *spp.* species

### Knowledge, experience, and perceptions related to helminth infections

Due to the significant burden of multiple helminth infections in riverside villages compared to inland villages, further stratified analysis was done to find out the differences between those two categories in terms of awareness, reported experience of intestinal helminth infections and knowledge of mode of infection, specific symptoms and preventive measures, and reported hand hygiene and food habits (Table 4).

**Table 4:** Knowledge of intestinal worms by study site, Shwegyin Township, Myanmar, 2017

Characteristic	Inland		Riverside		Total	
	No.	%	No.	%	No.	%
Awareness	(*n* = 101)		(*n* = 138)		(*n* = 239)	
Ever heard about intestinal worms*	77	76.2	124	89.9	201	84.1
Ever had intestinal worms*	50	49.5	56	40.6	106	44.4
Mode of infection of intestinal worms wormsϮ	(*n* = 47)		(*n* = 102)		(*n* = 149)	
Eating fly-contaminated food*	29	61.7	79	77.5	108	72.5
Eating with dirty hands*	21	44.7	81	79.4	102	68.5
Playing with soil*	14	29.8	75	73.5	89	59.7
Eating unwashed raw vegetables or meat*	13	27.7	73	71.6	86	57.7
Biting dirty fingernails*	12	25.5	71	69.6	83	55.7
Playing in dirty places*	9	19.1	67	65.7	76	51.0
Walking barefoot*	8	17.0	62	60.8	70	47.0
Through mosquito bite (NS)	6	12.8	26	25.5	32	21.5
Perceived sickness by intestinal worms*	41	40.6	104	75.4	145	60.7
Specific symptoms noted	(*n* = 42)		(*n* = 99)		(*n* = 141)	
Abdominal colic (NS)	37	88.1	87	87.9	124	87.9
Diarrhea*	17	40.5	62	62.6	79	56.0
Poor appetite*	10	23.8	51	51.5	61	43.3
Feeling tired (NS)	14	33.3	45	45.5	59	41.8
Weight loss*	9	21.4	45	45.5	54	38.3
Fever (NS)	10	23.8	40	40.4	50	35.5
Prevention of intestinal worm infection*	(*n* = 53)		(*n* = 109)		(*n* = 162)	
Washing fruits before eating	30	56.6	89	81.7	119	73.5
Washing hands before eating	24	45.3	84	77.1	108	66.7
Washing hands after toilet	21	39.6	85	78.0	106	65.4
Using latrine	17	32.1	73	67.0	90	55.6
Keeping up personal hygiene	12	22.6	77	70.6	89	54.9
Covering food	11	20.8	72	66.1	83	51.2
Not playing in the soil	10	18.9	69	63.3	79	48.8
Not playing in the dirt	11	20.8	67	61.5	78	48.1
Wearing slippers all the time	9	17.0	69	63.3	78	48.1

*NS* not significant

*P < 0.05

Only 10% of the respondents had never heard of intestinal helminths (11.9% from the inland villages and 8.7% from the riverside villages; *P* < 0.05). Whatsoever, around 44% of them had experienced intestinal helminth infections (49.5% from inland and 40.6% from riverside villages; *P* < 0.05; Table 4). Even though 201 respondents had ever heard of intestinal worm infections, only 149 of them (74%) could report the specific modes of infection that required attention. The knowledge of the modes of infection of intestinal worms was analyzed. Some of the respondents could cite eating fly-contaminated food (73%), playing in dirty places (51%), and walking barefoot (47%), but 22% of them reported mosquito bite which was a misperception. The most commonly cited symptom was abdominal colic (87.9%) followed by diarrhea and poor appetite. As regards the preventive measures, over 70% of respondents mentioned “washing fruits before eating” followed by “washing hands before eating” (66.7%) and “washing hands after toilet” (65.4%) as possible ways to prevent intestinal helminth infections. However, less than 60% of respondents in the combined sample recognized the use of latrines for defecation, observing personal hygiene, and proper covering of food could prevent intestinal helminth infections and nearly 50% mentioned avoidance of playing in the ground and dirt and wearing slippers as preventive measures. Surprisingly, the knowledge of respondents from the riverside villages related to the modes of infection and preventive measures was significantly higher than those from inland villages regardless of their higher level of education attainment as shown in Table 1. This finding indicated gaps between knowledge and their real practice such as sanitary and hygienic practices and regular deworming (Table 4).

### Translation of research evidence, challenges, and potential strategies addressed

The research team transferred the evidence-based findings as the estimated prevalence > 25% of STH and < 5% of other zoonotic helminths which were especially common in pre-school-age children, school-age children, and seasonal gold panning workers despite annual mass drug administration for filariasis control, school deworming, and water, sanitation, and hygiene (WASH) interventions. Following salient findings emerged:

1. The information on significantly higher infection rates among community risk groups at riverside villages compared to inland villages was stressed and validated during the interactive dialogue session.
2. Moreover, BHS discussed their opinions towards best practices including food and deworming habits among community members in relation to helminth infections.
3. The interactive dialogue session also elucidated poor socio-economic status linking to poor personal hygiene of children with high chance of helminth infections with more than one species and other disadvantaged groups such as children from families of mobile/migrant workers, children who did not go to school and missed the opportunity of school deworming programs, and seasonally mobile gold panning workers with an exposure to dual risk of poor environmental conditions both in their villages and at their worksites.
4. The dialogue further highlighted the role of insanitary latrines and improper disposal of children’s excreta in spreading helminth infections in the locality. The innovative ideas/strategies recapitulated by BHS included the integration of health talks during the sessions for small agricultural loans and to harness advocacy with water, sanitation, and hygiene interventions.

## Discussion

This study highlighted the emergence of neglected zoonotic helminthiasis in Myanmar and its associated determinants that significantly varied by geographical location. The overall prevalence of infection for any helminth by K-K method was 24% in this study which was more or less similar to the previous study focusing pregnant women during 2012–2014 in the same study area [13]. Another study conducted in three townships of Yangon Region during 2013 reported the overall prevalence of helminth infection as 21% (303/1443) [28]. Above all, the likelihood of prevalence of helminth infections > 20% in the study sites consistent to other studies pointed out the need for transmission elimination plans including mass deworming. The success may depend upon the increased investment to address the research gaps through the appropriate use of sensitive diagnostic tools to detect persistent infections and polyparasitism apart from the conventional K-K method [29, 30].

In this study, the rate of helminth infections of all species (STH together with zoonotic helminths) in riverside villages was three times higher than that in inland villages probably due to frequent flooding and unsanitary conditions. The adequate health sector response might depend on community engagement to take part in sustainable actions towards preventive interventions for helminth infections [31]. In this scenario, riverside villages are usually flooded two times a year and more humid and muddy soil could form a favorable environment for the helminth development. The environmental degradation occurred, but free-living stages of some STHs are highly resistant leading to persistence in the soil for years. The development of helminth in the environment mainly depends on humidity before infecting the host [32]. In that case, the success of control and elimination programs for helminthic infections depends on optimum treatment coverage with mass drug administration (MDA) twice per year according to WHO guidelines apart from school deworming program. However, community involvement and participation as a community buy-in needs further encouragement to prevent reinfections apart from MDA [33].

Moreover, study participants of riverside villages reported ingesting raw vegetables and unwashed fruits more than inland villages that required attention to promoting health literacy as one of the components of integrated preventive health interventions [29, 34]. Taeniasis was detected in 0.7% (5/698) of the total population. For trematodiasis, *Schistosoma* spp. was detected in 2% (6/298) of inland villages and 4% (17/400) of riverside villages. Very few cases of liver fluke infections (*Opisthorchis viverini*/*Clonorchis sinensis*) were also detected in 1.7% (5/298) of inland and 2.5% (10/400) of riverside villages. These trematodes were not previously reported and perceived as uncommon in the locality. The previous work in Shwegyin Township in 2016 through molecular verification has already confirmed the presence of *Schistosoma* spp. in 8 out of 204 samples [35]. Those infections with an estimated prevalence of less than 5% in the community should pay attention to individual treatment with Praziquantel [36]. Alterations in the environment in recent years due to climate change and natural disasters, together with increasing seasonal mobility of migrant workers, further increase the risk of zoonotic helminthiases. The detected helminth infections in this study: *Opisthorchis viverini*, *Clonorchis sinensis*, and *Schistosoma* spp. though negligible were of snail-borne in nature which was commonly found in freshwater. Accurate descriptions of the geographical distribution of infection and co-infection could facilitate the implementation of sustainable and cost-effective control measures [37].

Nearly half of the gold panning workers were helminth infected in this study though the sample was small. Shwegyin has several gold panning sites, and although seasonal, their nature of work is to stay a long time underneath the shallow water superimposed by poor environmental conditions. It is critical to promote an access to health services for individual diagnosis and treatment as one of the strategies for a large-scale elimination of the infection reservoir. Furthermore, favorable interventions should focus on improving sanitation, providing access to clean water, disposing adequately of excreta and solid waste, and facilitating adequate housing and health education [31]. High prevalence of helminth infections in riverside villages with poor sanitary conditions was validated by BHS during the interactive dialogue session. Especially in flood-prone areas, householders failed to follow the guidelines to construct/re-construct sanitary latrines which were consistent with other studies [38, 39] and challenges remained in disseminating health messages for community engagement.

### Strengths and limitations

This is the first study reported in Myanmar focusing on the significant impact of annual flooding on prevalence and intensity of soil-transmitted helminth infections and the emergence of other zoonotic helminths in rural areas. The following policy and programmatic implications are noted at vulnerable sites concerning the evidence-based findings during the interactive dialogue session with basic health staff that might facilitate:

1. To determine which programs would be most favorable to the integration of deworming activities for at-risk groups
2. To assist in identifying partners for implementation of integrated interventions and to encourage community buy-in through health literacy promotion efforts

However, owing to funding constraints and time and resources limitations, only one study township was selected. The collection and examination of a single stool sample might miss estimating the actual prevalence of neglected intestinal helminth infections due to the intermittent shredding of helminth eggs. In addition, infection intensity in terms of light, moderate, and heavy infections could not be determined. The ownership of sanitary latrines in study households is subjected to false information due to the reliance on self-reported data without any observations, thereby leading to insignificant associations with multiple helminth infections. Being a cross-sectional nature, the study could not determine the varying intensity and persistent infections at multiple time points.

## Conclusions

The emerging evidence of neglected zoonotic helminths required attention to introduce the periodic mopping-up and the “selective deworming plan” for vulnerable groups to cover the missed targets. To provide comprehensive knowledge translation, the better understanding of geographical burden and environmental and social ecology of emerging neglected zoonotic helminthiasis is imperative. Further multidisciplinary research to confirm the intermediate hosts and vectors in the environment is essential for adequate surveillance and response actions.

## Additional file

Additional file 1: The map of Shwegyin Township showing the location of four study villages. (DOCX 177 kb)

## Abbreviations

BHS: Basic health staff
DALYs: Disability-adjusted life years
FBT: Food-borne trematodes
K-K: Kato-Katz method
Pre-SAC: Pre-school-age children
STH: Soil-transmitted helminths
THD: Township Health Department
