# Supplementary figures and images for: Emerging neglected helminthiasis and determinants of multiple helminth infections in flood-prone township in Myanmar
Source: Trop Med Health. 2019 Jan 4;47:1. doi: 10.1186/s41182-018-0133-6 (PMC6318856; doi:10.1186/s41182-018-0133-6)

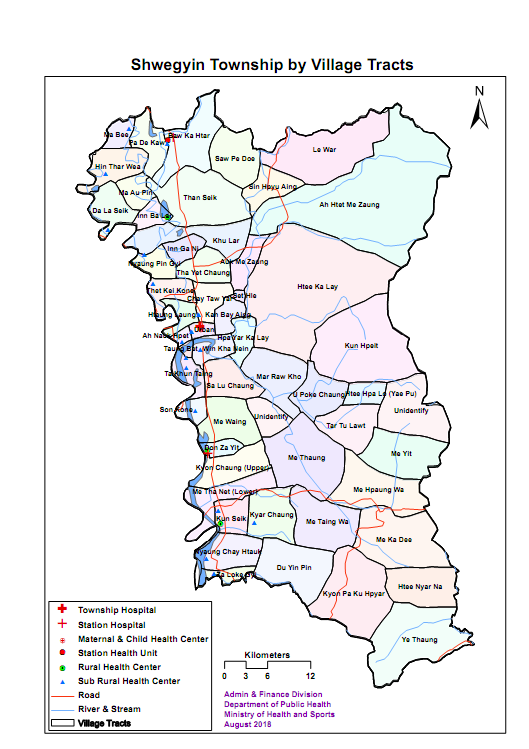


Inland

villages

Riverside villages

Supplement: Supplementary file 1 — The map of Shwegyin Township showing the location of four study villages. (DOCX 177 kb) [file 41182_2018_133_MOESM1_ESM.docx]
